# Classical Photoreceptors Are Primarily Responsible for the Pupillary Light Reflex in Mouse

**DOI:** 10.1371/journal.pone.0157226

**Published:** 2016-06-13

**Authors:** Varsha Jain, Ipsit Srivastava, Shriya Palchaudhuri, Manvi Goel, Sumit K. Sinha-Mahapatra, Narender K. Dhingra

**Affiliations:** National Brain Research Centre, Manesar (Gurgaon), Haryana, India 122051; Morehouse School of Medicine, UNITED STATES

## Abstract

Pupillary light reflex (PLR) is an important clinical tool to assess the integrity of visual pathways. The available evidence suggests that melanopsin-expressing retinal ganglion cells (mRGCs) mediate PLR—driven by the classical photoreceptors (rods and cones) at low irradiances and by melanopsin activation at high irradiances. However, genetic or pharmacological elimination of melanopsin does not completely abolish PLR at high irradiances, raising the possibility that classical photoreceptors may have a role even at high irradiances. Using an inducible mouse model of photoreceptor degeneration, we asked whether classical photoreceptors are responsible for PLR at all irradiances, and found that the PLR was severely attenuated at all irradiances. Using multiple approaches, we show that the residual PLR at high irradiances in this mouse was primarily from the remnant rods and cones, with a minor contribution from melanopsin activation. In contrast, in rd1 mouse where classical photoreceptor degeneration occurs during development, the PLR was absent at low irradiances but intact at high irradiances, as reported previously. Since mRGCs receive inputs from classical photoreceptors, we also asked whether developmental loss of classical photoreceptors as in rd1 mouse leads to compensatory takeover of the high-irradiance PLR by mRGCs. Specifically, we looked at a distinct subpopulation of mRGCs that express Brn3b transcription factor, which has been shown to mediate PLR. We found that rd1 mouse had a significantly higher proportion of Brn3b-expressing M1 type of mRGCs than in the inducible model. Interestingly, inducing classical photoreceptor degeneration during development also resulted in a higher proportion of Brn3b-expressing M1 cells and partially rescued PLR at high irradiances. These results suggest that classical photoreceptors are primarily responsible for PLR at all irradiances, while melanopsin activation makes a minor contribution at very high irradiances.

## Introduction

Broadly, classical photoreceptors (rods and cones) produce image-forming vision, while melanopsin-expressing retinal ganglion cells (mRGCs) are considered to be responsible for non-image-forming vision. However, this distinction has blurred with several recent discoveries. For example, classical photoreceptors are also involved in non-image-forming functions, such as circadian activity and pupillary light reflex (PLR), and mRGCs also contribute to pattern vision [1–9]. The majority of the work designed to unravel the differential roles of classical photoreceptors and mRGCs has been carried out using genetically-engineered mice. Since genetic alterations—especially when introduced during development—can result in a variety of compensatory biological responses, the results from using such animal models may be difficult to interpret [5,10–15]. For example, knocking out TRβ (thyroid hormone receptor) gene, which is involved in the development of m-cones, results not only in lack of m-opsin but also in upregulation of s-opsin and melanopsin [15]. Since mRGCs are known to receive signals from photoreceptors [16–20], loss of photoreceptors during development could potentially cause compensatory changes in mRGCs, making it difficult to understand their specific contributions to vision.

Loss of photoreceptors during development, caused by genetic mutation (eg, in PDE6β^rd1^, rd/rd-cl or gnat^-/-^;cnga3^-/-^ mice), results in elimination of PLR at low irradiances but leaves it intact at high irradiances [1,7,21–23]. This suggested that photoreceptors are responsible for PLR only at low irradiances. Knocking out the melanopsin gene in these mice (PDE6β^rd1^;Opn4^-/-^ or gnat^-/-^;cnga3^-/-^;Opn4^-/-^) eliminates PLR at all irradiances, suggesting that melanopsin is responsible for PLR at high irradiances [1,22]. However, this is inconsistent with the observations that knocking out the melanopsin gene (Opn4^-/-^) or acutely blocking the melanopsin activation with a specific antagonist in an otherwise normal mouse causes only 10%–15% reduction in the high-irradiance PLR [1,21,22,24].

Here, we explored the possibility that classical photoreceptors are responsible for PLR at all irradiances. Since classical photoreceptors provide input to mRGCs, it was possible that loss of classical photoreceptors during development leads to a compensatory response in mRGCs which then take over the PLR function at high irradiances. To test these hypotheses, we measured PLR in adult mouse in which classical photoreceptor degeneration was induced pharmacologically after normal retinal development; we predicted that unlike in rd1 mouse the PLR would be attenuated at all irradiances. To look for the potential compensatory cellular response in rd1 mouse, we studied Brn3b-expressing mRGCs which have been shown to mediate PLR [25].

## Materials and Methods

### Animals and Drug Injections

All experiments reported here were approved by the Institutional Animal Ethics Committee of the National Brain Research Centre, India. Adult (8–12 weeks) wild-type (C57BL/6J) and rd1 mice (CBA/J) were used. In addition, wild-type pups (P-8 to P-12) and pregnant mice were used for some experiments (see more details in Results). Mice were obtained from Jackson Laboratory (Bar Harbor, USA) and bred locally at the animal facility of the National Brain Research Centre, India. All animals were maintained on a 12-hour light:dark cycle with an average ambient daylight of approximately 200 lux (measured with an IL1400 photometer, International Light Technologies, Peabody, MA).

Some adult male mice (both wild-type and rd1) received a single injection of N-methyl-N-nitrosourea (MNU; 65 mg/kg in 0.05% acetic acid; i/p; Sigma-Aldrich), sodium iodate (NaIO_3_; 50 mg/kg; i/v; Sigma-Aldrich), or both MNU and NaIO_3_ [26,27]. MNU was also injected in some wild-type mice during development, either postnatally (P-8 to P-12; 60 mg/kg; i/p) or prenatally (in pregnant mothers at E-16; 5 mg/kg; i/p) [28]. Similarly, NaIO_3_ was injected in some wild-type pups at P-8 (60 mg/kg; i/p) [26].

To block melanopsin activity, we injected in some animals a specific melanopsin antagonist (1-(2,5-dichloro-4-methoxy-benzenesulfonyl)-piperdine (AA41612; 10 mg/kg, i/p; Princeton BioMolecular Research, Inc., Monmouth., NJ) [24]. The drug was prepared in dimethyl sulfoxide (Sigma-Aldrich) and phosphate-buffered saline (PBS) containing 18.2% hydroxypropyl-beta-cyclodextrine and 0.009M methane sulphonic acid (Sigma-Aldrich).

### Pupillary Light Reflex

The PLR was recorded between Zeitgeber Time 3 (ZT3) and ZT9 (ZT0: light turning on at 6 am) [7]. A mouse was dark adapted for 1 hour, lightly anesthetized with ketamine (50 mg/kg) and xylazine (5 mg/kg), and placed on a platform in a dark room. The light sedation was given to avoid using restraints, which can cause variable magnitude of anxiety and therefore variability in the measured PLR [7,29]. Consensual PLR was recorded using an infrared video camera (frame rate 30 Hz; DCR-HC96, Sony, Japan). White light from a 100 W halogen bulb was shone in one eye through a light guide connected to the lamp source (color temperature, 3100K; LG-PS2, Olympus, Japan) while recording the pupil response from the other eye. A custom-made barrier blocked the light from falling on the recorded eye. Neutral density filters (Olympus, Japan) were used to cover approximately 8 log units of irradiance (10^−4^ μW/cm^2^ to 2.8 x 10^4^ μW/cm^2^). Following the baseline recording for 60 sec in dark, PLR was recorded at different light intensities, presented in increasing order of intensity. Each stimulus was presented for 20 sec, which was followed by 100 sec of dark adaptation. The PLR in mice which received a melanopsin antagonist was recorded before the drug injection and again between 20 and 30 min after the injection [24]. For these animals, since the 10-min window was not sufficient to record the PLR at all irradiances, some of the irradiances were skipped, but we covered the full range of irradiances used for other animals.

The video frames were analyzed using imageJ software (NIH); the pupil boundary was marked using the *circle* tool in imageJ and the area within the boundary computed. The pupil constriction at a given light intensity was calculated as percentage change from the baseline pupil area. The initial constriction, which occurred at 2–3 sec after the stimulus onset, was taken as the transient pupil response. The constriction during the last 5 seconds of stimulus presentation (mean of 5 values at 1-second intervals during 16^th^ to 20^th^ second after the stimulus onset) was taken as sustained pupil response. The sustained pupil constriction is presented as PLR, unless noted otherwise.

### Characterization of primary antibodies

The primary antibodies used here are described in Table 1. The Brn3a antibody recognizes a 47 kDa band in Western blot (manufacturer’s specification). The specificity of the Brn3b antibody was confirmed by preadsorption with the appropriate Brn3b peptide (SC-6026P, Santa Cruz Biotechnology) (not illustrated). The melanopsin antibody has been extensively used previously. The specificity of this antibody has also been verified by the lack of immunoreactivity in melanopsin knockout mouse [30,31]. The melanopsin antibody used here for Western blotting (Table 1) detects two bands: one for glycosylated melanopsin at 85 kDa and the other for unglycosylated melanopsin at 53 kDa (manufacturer’s specifications) [32]. The β-tubulin antibody, which was used for loading control in Western blotting, detects a single band at 55 kDa (manufacturer’s specifications) [33].

**Table 1:** List of primary antibodies.

Antigen	Immunogen	Source	Species, type	Dilution
Brn3a	1–109 N-terminal amino acidsof mouse Brn3a	Santa Cruz Biotechnology;#sc-8429	Mouse, monoclonal	1:250
Brn3b	397–410 C-terminal amino acids(QRQKQKRMKYSAGI) of human Brn3b	Santa Cruz Biotechnology; #sc-6026	Goat, polyclonal	1:250
Melanopsin	a synthetic peptide consisting of 1–15 N-terminal amino acids (MDSPSGPRVLSSLTQ)of mouse melanopsin	Advanced Targeting Systems; #UF006	Rabbit, polyclonal	1:7500 (immunohistochemistry)
Melanopsin	A synthetic peptide consisting of amino acids 1–19 (KMNSPSESRVPSSLTQDPSF) of rat melanopsin	Abcam; ab19306	Rabbit; polyclonal	1:2000 (Western blotting)
M-Opsin	Recombinant human red/green opsin (last 38 C-terminal amino acids)	Chemicon; # AB5405	Rabbit, polyclonal	1:500
S-Opsin	Recombinant human blue opsin (last 42 C-terminal amino acids)	Chemicon; #AB5407	Rabbit polyclonal	1:1000
Rhodopsin	Bovine rhodopsin (clone: Rho 1D4; last 9 C-terminal amino acids)	Chemicon; # MAB5356	Mouse, monoclonal	1:5000
β-Tubulin	Tubulin derived from rat brain	Sigma; #T4026	Mouse, monoclonal	1:16000

To measure the extent and pattern of rod and cone loss, retinal flatmounts were immunolabeled for rhodopsin (rods), m-opsin (m-cones), or s-opsin (s-cones). The antibody against rhodopsin showed a strong labeling of the outer segments and weak labeling of the inner segments and the cell bodies of rods [27], confirming the antibody specificity. The antibodies against m-opsin and s-opsin detect 40 kDA and 39 kDa bands, respectively, in Western blotting (manufacturer’s specification). Since long wavelength (red) cones are absent in mouse, the red/green opsin antibody labeled only green cones.

### Immunohistochemistry

Retinal flatmounts were prepared from the mice used for PLR measurements and immunolabeled as described previously [9]. Briefly, eyes were removed after cervical dislocation, and eyecups were fixed in 4% paraformaldehyde (PFA; pH 7.4) at 4°C for 1 hour. Retina was isolated from the eyecup, incised radially or cut into 3–4 radial pieces, and flattened on a membrane filter (Millipore, Billerica, MA). For double labeling with melanopsin and Brn3b antibodies, the flatmounts were first incubated for 1 hour in a blocking solution containing 10% normal donkey serum (NDS), 3% bovine serum albumin (BSA), and 0.3% Triton X-100 in PBS (pH 7.4). The samples were then incubated in a mixture of antibodies against melanopsin and Brn3b at 4°C for 3 days. After washing in PBS for 5 x 5-min, the samples were incubated in the appropriate secondary antibody (donkey anti-rabbit conjugated with Alexa Fluor 488 [1:500] for melanopsin and donkey anti-goat conjugated with Alexa Fluor 594 [1:500] for Brn3b; both from Molecular Probes, Eugene, OR) for 1 hour at room temperature. The samples were washed again in PBS for 5 x 5-min and mounted in Vectashield containing 4-,6-diamidino-2-phenylindole (DAPI; Vector Laboratories Inc., Burlingame, CA).

To assess the effect of photoreceptor loss on density of non-mRGCs, retinal flatmounts of adult wild-type, rd1 and MNU-injected mice were immunolabeled for Brn3a [9]. The blocking solution was same as mentioned above, and the secondary antibody was donkey anti-mouse conjugated with Alexa Flour 488 (1:500).

To quantify the number of rods, m-cones and s-cones, we used rhodopsin, m-opsin and s-opsin antibodies, respectively. The retinal wholemounts were incubated for 1 hour in a blocking solution containing 3% NDS, 1% BSA and 0.3% Triton X-100 in PBS (pH 7.4). The samples were then incubated overnight in respective primary antibodies at 4°C. After washing in PBS for 5x 5-min, the samples were incubated in the appropriate secondary antibody (donkey anti-mouse conjugated with Alexa Fluor 488 (1:500) for rhodopsin; donkey anti-rabbit conjugated with Alexa Fluor 488 (1:500) for m-opsin; donkey anti-rabbit conjugated with Alexa Fluor 488 (1:500) for s-opsin (all from Molecular Probes, Eugene, OR) for 1 hour at room temperature.

### Imaging and morphometric analyses

The imaging and analyses were performed as described previously [9]. Briefly, the images of immunolabeled flatmounts were captured on an epifluorescent microscope (AxioImager.Z1; Carl Zeiss, Gottingen, Germany) fitted with a CCD camera (AxioCam MRm). The microscope was equipped with ApoTome grid projection system, which allowed capturing images at higher contrast and enhanced optical resolution in z-axis. M1 and non-M1 type of mRGCs were identified based on their dendritic stratification in the IPL. For tracking the dendrites of melanopsin cells, several 1-μm thick serial optical sections were captured in z-axis to cover the GCL and the IPL. The images were taken such that the relatively bright (likely M1) cells were near the center of the field. If the dendrites of these cells stratified in outer IPL, we then determined whether any of the other dendrites stratified in inner IPL, to differentiate between M1 and M3 cells. The remaining cells were identified as M2 or other non-M1 cells. Once a melanopsin cell was identified as M1 or non-M1 type, we examined each cell for Brn3b expression. For this, we enhanced the image contrast to be able to detect even the very faint Brn3b expression [9]. Total mRGCs, M1 and non-M1 mRGCs, and Brn3b-expressing mRGCs were counted and density was computed.

To quantify the number of rods and cones, images of labeled retinas were captured using a confocal (LSM 510 Meta, Carl Zeiss) or the epifluorescent microscope. For cones, overlapping images were captured, and stitched using Photostitch software (Canon, USA) to generate a montage of the whole retina. The m-cones and s-cones were counted in these montages using ImageJ. For rods, we captured non-overlapping images to cover a large part of a retina, and measured the labeling intensity. All z-sections with any rhodopsin signal in a frame were merged while averaging the labeling intensity at each pixel. The integrated density of rhodopsin labeling was computed by summing intensity values of all the pixels after subtracting the background.

### Western blotting

Western blotting was performed as described earlier [34]. Briefly, a 20 μg of each tissue sample was electrophoresed in 9% SDS-PAGE and then transferred to a polyvinylidene fluoride (PVDF) membrane. The blots were incubated for 1 hour at room temperature in a blocking solution (5% BSA in Tris-buffered saline with 0.1% Tween-20 (TBST), pH 7.4) and then in melanopsin antibody (1:2000) overnight at 4°C. β- tubulin was used as loading control. This was followed by 3x 10-min washes in TBST, followed by incubation in the secondary antibody at room temperature for one hour. The secondary antibodies used were horseradish-peroxidase-conjugated anti-rabbit and anti-mouse (Vector Laboratories, Burlingame, USA) for melanopsin and β-tubulin, respectively (both 1:4000). Blots were washed again 3x 10-min in TBST. The signals were developed using enhanced chemiluminescence (ECL; Millipore, Billerica, USA) and captured on GelDoc (Syngene Chemi Genius 2 Bio imaging system, Cambridge, UK). Densitometric analysis was performed using ImageJ software. The grayscale image was ‘‘inverted” and the background was subtracted using the rolling ball radius method in imageJ. The data was expressed as optical density (OD).

### Quantitative Real-Time PCR

Adult wild-type (n = 11), rd1 (n = 11) and MNU-injected mice (14 days post-injection; n = 11) were used to study the melanopsin expression levels. Animals were sacrificed by cervical dislocation at ZT 14, eyes removed, hemisected, and retina isolated and processed for RNA isolation. Retinas were collected in 250 μl of Trizol (Sigma-Aldrich) and homogenized using a homogenizer (IKA-Werke). The RNA was then isolated using the phenol-chloroform extraction method. The cDNA was synthesized from 1ug of total RNA, using Transcriptor First Strand cDNA Synthesis Kit (Roche, Cat.No: 04896866001) as per kit protocol. The primers for melanopsin and 18S ribosomal RNA were designed using NCBI and obtained from Sigma-Aldrich. The annealing temperature was standardized using end point Reverse Transcriptase PCR. The primer sequences are as follows:

18S ribosomal RNA:

Forward: GAGGGAGCCTGAGAAACGG

Reverse: GTCGGGAGTGGGTAATTTGC

Melanopsin:

Forward: CCCAGCACCTGCCTTGCCTT

Reverse: TTCTGTGTCTGTCCAGCCCACT

The Real Time PCR reactions were performed in PRISM 7500 Sequence Detection system (Applied Biosystems, CA, USA) using Power SYBR Green PCR Master Mix (Applied Biosystems, CA, USA) as per the protocol. Each reaction was done in duplicate and 18S r-RNA was used as a loading control. 500ng of cDNA was used as the starting material for all the samples. The PCR conditions were: 95°C for 10 min (1 cycle) for activation of Taq polymerase enzyme, followed by 40 cycles of 95°C for 20s (denaturation), 58°C for 30s (annealing temperature) and 72°C for 40s (extension). The data collection was done at extension phase. To verify that the efficiency of primer pairs, without homo- and hetero-dimer formation and producing only a single product, a dissociation protocol was performed. The data was collected using sequence detector software (SDS) and analyzed using ΔΔCT method (relative quantification).

### Statistical Analyses

Mixed design analysis of variance (ANOVA) with repeated measures, two-way ANOVA with repeated measures, one-way ANOVA with or without repeated measures were employed to analyze the data in Sigmaplot (Systat Software Inc., CA, USA) or SPSS softwares (IBM, NY, USA). A p-value of less than 0.05 was considered statistically significant. The data on proportion of M1 cells expressing Brn3b were transformed for statistical comparison using arcsine transformation. A two-tailed (paired or unpaired) t-test was used for some comparisons.

## Results

### PLR was severely attenuated at all irradiances in mice in which classical photoreceptor degeneration was induced during adulthood

**Fig 1:**
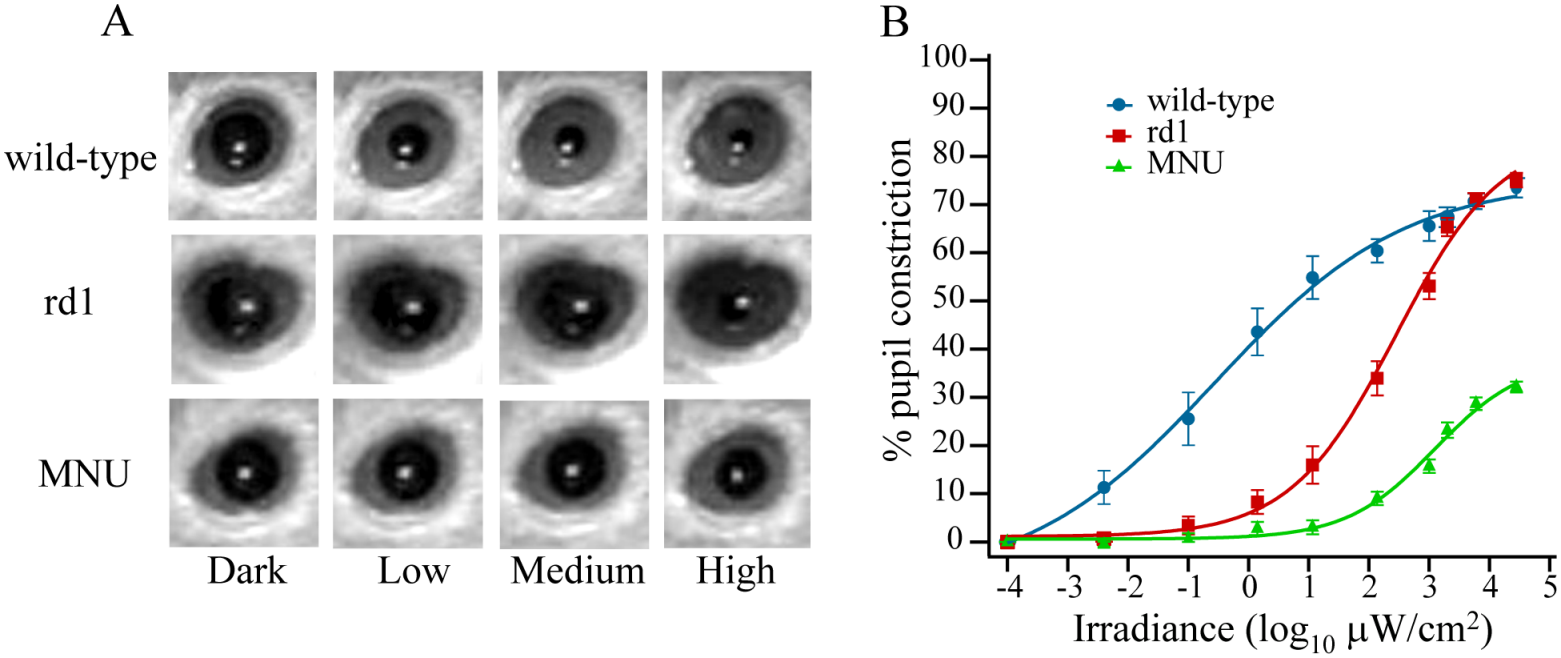
PLR was severely attenuated at all irradiances in mice in which photoreceptor loss was induced with MNU during adulthood. **A)** Representative pupil images from three groups of mice, showing sustained pupil response in dark, and at low (0.1 μW/cm^2^), medium (10 μW/cm^2^), and high (2.8 X 10^4^ μW/cm^2^) irradiances. **B)** Percent pupil constriction (mean ± SE) as a function of irradiance. A mixed design ANOVA with repeated measures on irradiance showed significant differences among the three groups (F_2,21_ = 97.6; *P* <<0.0001). A Bonferroni post hoc test showed all three groups were different from each other at *P* <<0.0001. A pairwise comparison at each irradiance revealed that PLR in MNU-injected mouse was significantly lower than wild-type at all irradiances used here (*P* = 0.0005 at 4x10^-3^ μW/cm^2^ and *P* << 0.0001 at higher irradiances), while it was lower than rd1 mice at 10 μW/cm^2^ (*P* <0.05) and higher irradiances (*P* <<0.0001). The PLR in rd1 mouse was similar to wild-type at irradiances >10^3^ μW/cm^2^ (*P* >0.05) (n = 8 mice for all groups).

In wild-type mouse, the sustained pupil constriction increased with irradiance, first rapidly (~25% constriction at 0.1 μW/cm^2^ and ~45% at ~1 μW/cm^2^) and then slowly (~55% constriction at 10 μW/cm^2^ and ~70% at ~10^4^ μW/cm^2^) (Fig 1A and 1B). The rd1 mouse showed negligible pupil constriction at irradiances up to 0.1 μW/cm^2^ and ~5% constriction at 1 μW/cm^2^, but the constriction increased rapidly beyond 1 μW/cm^2^ to approach the levels similar to those in wild-type. These observations in wild-type and rd1 mice are similar to earlier reports [7,21,22,35], and imply that classical photoreceptors are required for PLR at low irradiances.

In mouse in which classical photoreceptor degeneration was induced with MNU during adulthood, the pupil constriction at 7 days post-injection (dpi) was similar to rd1 mouse at lower irradiances (Fig 1A and 1B). However, unlike rd1 mouse, pupil constriction in MNU-injected mouse did not increase rapidly beyond 1 μW/cm^2^. Even at the highest irradiance used here (2.8 x 10^4^ μW/cm^2^), the pupil constriction in MNU-injected mouse was only ~30% (Fig 1B). We measured PLR in these mice for up to 6 months after MNU injection, and found that most of the reduction occurred during the first week (data not illustrated). These results implied that classical photoreceptors have a crucial role in PLR, not just at low, but also at high irradiances.

### Attenuation of PLR in MNU-injected mouse was attributable to loss of classical photoreceptors

**Fig 2:**
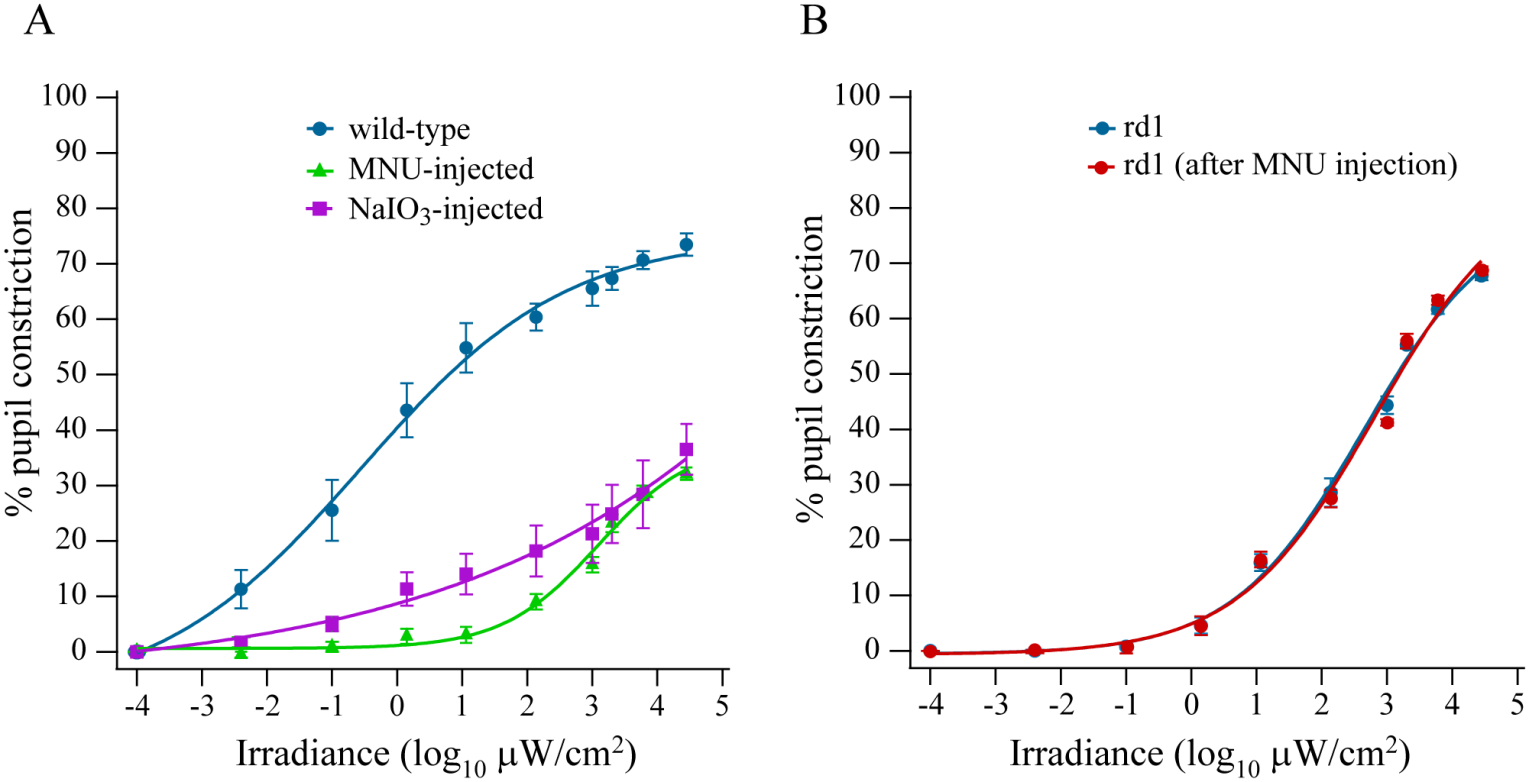
Attenuation of PLR in MNU-injected mice was attributable to loss of photoreceptors. **A)** NaIO_3_-induced photoreceptor loss led to severely attenuated PLR at all irradiances used here. At 7 days after NaIO_3_ injection, pupil constriction at the highest irradiance was ~35% (n = 6 mice). PLR data for wild-type and MNU-injected animals are replotted from Fig 1B for comparison. **B)** MNU did not affect PLR in rd1 mouse. PLR was recorded before and 7 days after MNU injection (two-way ANOVA with repeated measures; F_1,8_ = 0.02, *P* = 0.9; n = 5 mice).

One concern was that the attenuation in PLR in MNU-injected mouse could come from the drug directly affecting other retinal neurons, particularly the mRGCs. We addressed this with two experiments. First, we injected in some animals NaIO_3_, which is also known to induce classical photoreceptor degeneration [26]. The NaIO_3_-induced photoreceptor loss in adult mouse also led to severely attenuated PLR at all irradiances (Fig 2A). Second, we injected MNU in adult rd1 mouse. If MNU affected the mRGCs or other retinal neurons, the PLR in the MNU-injected rd1 mouse should also be significantly reduced at high irradiances. However, the PLR in rd1 mouse before and 7 days after MNU injection was similar at all irradiances (Fig 2B). Together, these results implied that the severely attenuated PLR in mice that were injected with MNU (or NaIO_3_) after normal retinal development was attributable to loss of classical photoreceptors, and not to any direct effect of the drug on mRGCs or other neurons in retina or other parts of the brain.

### Residual PLR at high irradiances in MNU-injected mouse was attributable primarily to remnant classical photoreceptors

If classical photoreceptors play a critical role in PLR at all irradiances, we wondered why there was any residual PLR in MNU-injected mouse at high irradiances (Fig 1B). One possibility was that some photoreceptors escaped MNU-induced degeneration and were responsible for the residual PLR. Alternatively, or additionally, the residual PLR could have originated from activation of melanopsin by high-irradiance stimuli. Using several approaches, we explored these possibilities.

**Fig 3:**
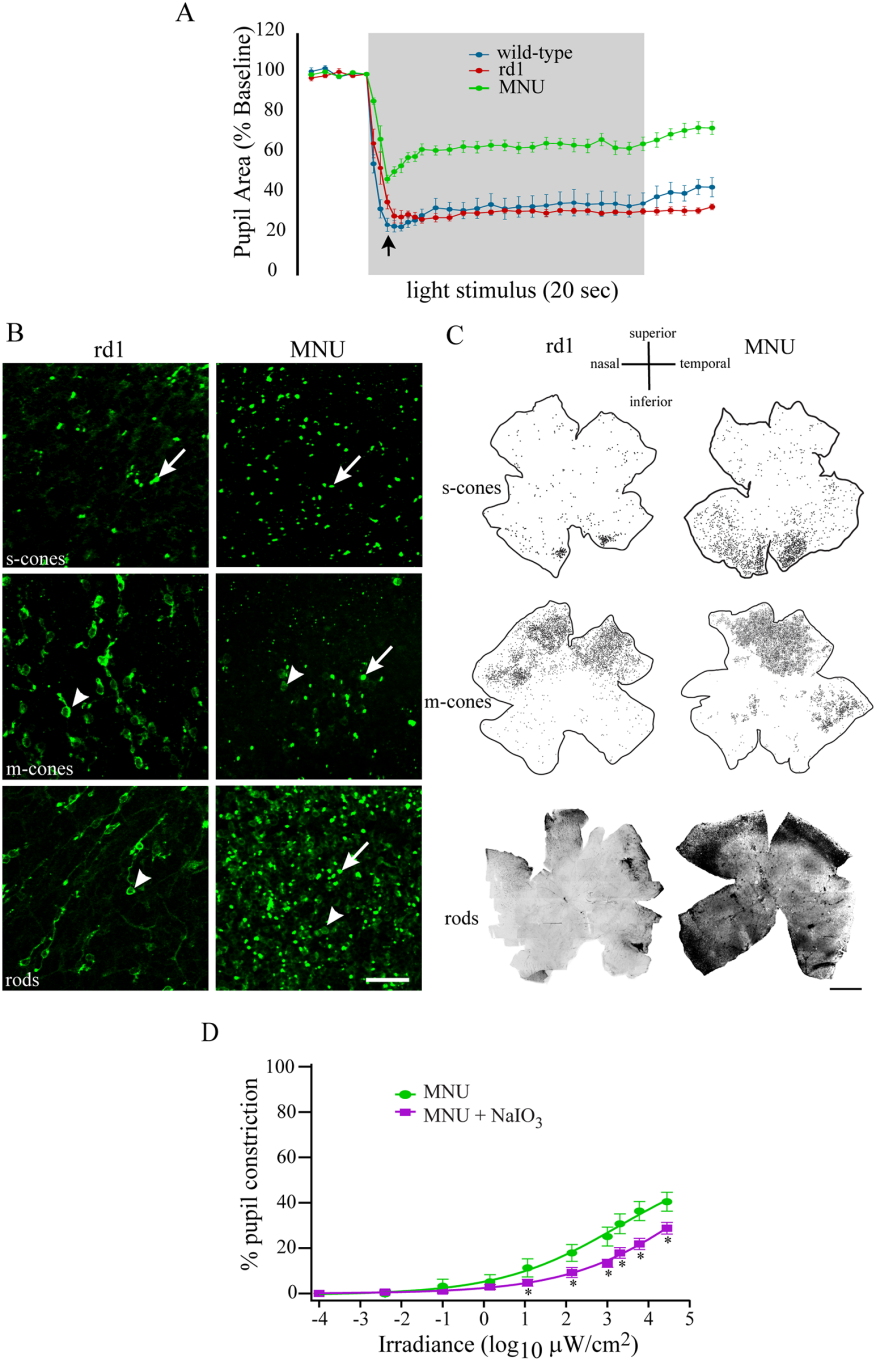
Residual PLR in MNU-injected mice was attributable primarily to remnant photoreceptors. **A)** Temporal profile of PLR (mean ± SE; n = 5 mice) at a high irradiance (6x10^3^ μW/cm^2^) for three groups of mice. MNU-injected (7 dpi) mice showed transient response ~2 sec after the stimulus onset (*arrow*), which was qualitatively similar to that in wild-type mice, whereas rd1 mice did not show any such transient response. **B)** Representative images of flatmounted rd1 (*left column*) and MNU-injected (*right*) mouse peripheral retinas, showing immunolabeling for s-opsin (*top row*; inferior retina), m-opsin (*middle*; superior retina) and rhodopsin (*bottom*; superior retina). S-opsin labeling was present as bright puncta (*arrows*), possibly representing remnant s-cone outer segments. M-opsin and rhodopsin labeling was present as puncta (*arrows*), as well as in cell somas (*arrowheads*). Scale bar: 20 μm. **C)** Schematics or montages of wholemount retinas labeled for s-opsin (*top row*), m-opsin (*middle*) and rhodopsin (*bottom*) showing the extent and pattern of photoreceptor loss in rd1 (*left*) and MNU-injected (*right*) mice. Each black dot in the schematics (top 2 rows) represents a remnant cone. To ensure that cones could be seen as distinctly as possible, we used smaller dot sizes where their density was too high. The number of rods in the MNU-injected sample was so high, especially in peripheral retina that we could not generate a schematic, and therefore show here the images of the montage from both rd1 and MNU-injected mice. Scale bar: 1 mm. **D)** Administration of NaIO_3_ in MNU-injected mice resulted in further reduction in PLR at high irradiances at 7 dpi. A two-way ANOVA with repeated measures on irradiance showed a significant difference between the two groups (F_1,45_ = 8.7; *P* = 0.032; n = 6 mice). A pairwise comparison using post hoc Holm-Sidak test revealed significant differences at irradiances ≥10 μW/cm^2^. *P <0.05.

First, we studied the transient pupil response in rd1 and MNU-injected mice. The transient pupil response is considered to originate from classical photoreceptors [7,18]. We found that wild-type and MNU-injected mice, but not rd1 mice, showed transient pupil response at approximately 2 sec after the stimulus onset (Fig 3A; *arrow*). Furthermore, MNU-injected mice appeared to show more rapid recovery at the stimulus offset than rd1 mice (Fig 3A). These observations suggested that the MNU-injected mouse had more remnant photoreceptors than rd1 mouse. This was also consistent with our finding that injecting MNU in rd1 mouse did not further reduce its PLR (Fig 2B).

We also compared the status of remnant photoreceptors in rd1 and MNU-injected mouse retinas directly by immunolabeling them for s-opsin, m-opsin, or rhodopsin. The number of puncta labeled for s-opsin in MNU-injected mouse (mean ± SE; 2620 ± 1092 per retina; n = 2 retinas) was approximately 5-fold higher than in rd1 mouse (521 ± 189; n = 2 retinas). However, the number of somas and puncta labeled for m-opsin in MNU-injected mouse (5744 ± 2269; n = 4 retinas) was generally similar to that in rd1 mouse (4247 ± 235; n = 2 retinas). In rd1 mouse, we found 2735 ± 790 (n = 2 retinas) rhodopsin-positive cell somas per retina. These somas were present mostly in the peripheral retina. However, the number of rhodopsin-positive entities in the MNU-injected sample was so high, especially in peripheral retina, that we could not count them reliably. Therefore, we measured the integrated density of rhodopsin labeling in retinal wholemounts, and found that it was approximately 20-fold higher in MNU-injected mouse (2.9 x 10^6^; n = 1 retina) than in rd1 mouse (1.6 x 10^5^; n = 1 retina) (Fig 3B and 3C).

Next, to understand whether the higher numbers of remnant classical photoreceptors in the MNU-injected mouse could account for the residual PLR, we administered NaIO_3_ (60 mg/kg; i/v) in some MNU-injected mice, expecting that this will lead to additional loss of classical photoreceptors and will further reduce the residual PLR. We found that NaIO_3_ indeed resulted in further reduction in PLR at high irradiances (Fig 3D). In some animals, we also injected multiple doses of MNU and found further reduction in PLR (data not shown). Together, these results suggested that there were more remnant classical photoreceptors in the MNU-injected mouse than in the rd1 mouse, and that they contributed to the residual PLR in the MNU-injected mouse.

### Melanopsin activation made a minor contribution to PLR at high irradiances

**Fig 4:**
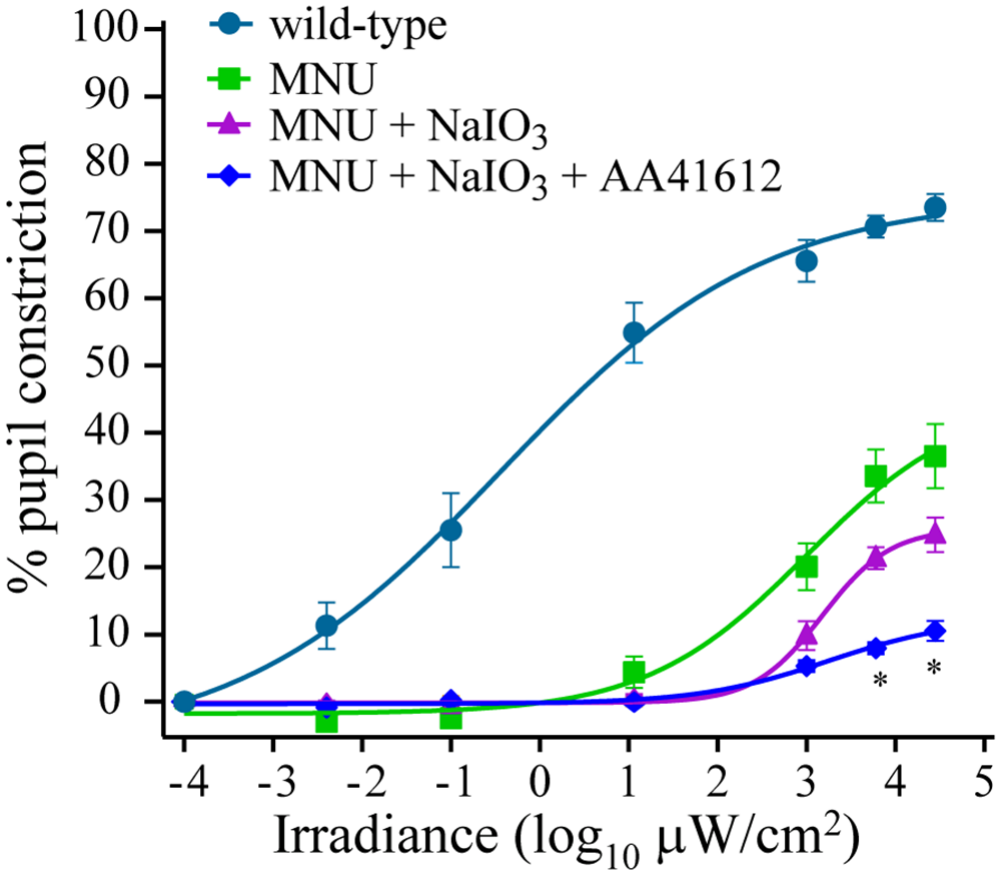
Melanopsin made a minor contribution to PLR at high irradiances. Three of the 6 mice shown in Fig 3D, which had both MNU and NaIO_3_ injected, were administered a melanopsin antagonist (AA41612). This resulted in further reduction in PLR at high irradiances. A two-way ANOVA with repeated measures showed a significant difference between the three groups (F_2,24_ = 31.3; *P* = 0.004). A post hoc Holm-Sidak test showed that injecting AA41612 resulted in significantly lower PLR at irradiances >10^3^ μW/cm^2^ compared with mice injected with MNU and NaIO_3_. PLR data for wild-type mice is replotted from Fig 1B for comparison.*P <0.05.

Our results from inducible models of classical photoreceptor degeneration suggested that melanopsin contribution to PLR was relatively minor (<30% at the highest irradiance used here; Figs 1B, 2A and 3D). To further investigate whether, and to what extent, melanopsin activation contributed to the high-irradiance PLR, we blocked melanopsin activity in the animals previously injected with MNU and NaIO_3_, using a recently-discovered melanopsin antagonist (AA41612, 10 mg/kg, i/p) [24]. This resulted in further reduction in PLR at high irradiances (>10^3^ μW/cm^2^) (Fig 4). Interestingly, even at the highest irradiance used here, the melanopsin antagonist reduced the PLR by <15% (Fig 4), a value that matches the magnitude of reduction in high-irradiance PLR in melanopsin knockout mouse [22].

### A higher proportion of M1 cells expressed Brn3b in rd1 mouse, but not in MNU-injected mouse

**Fig 5:**
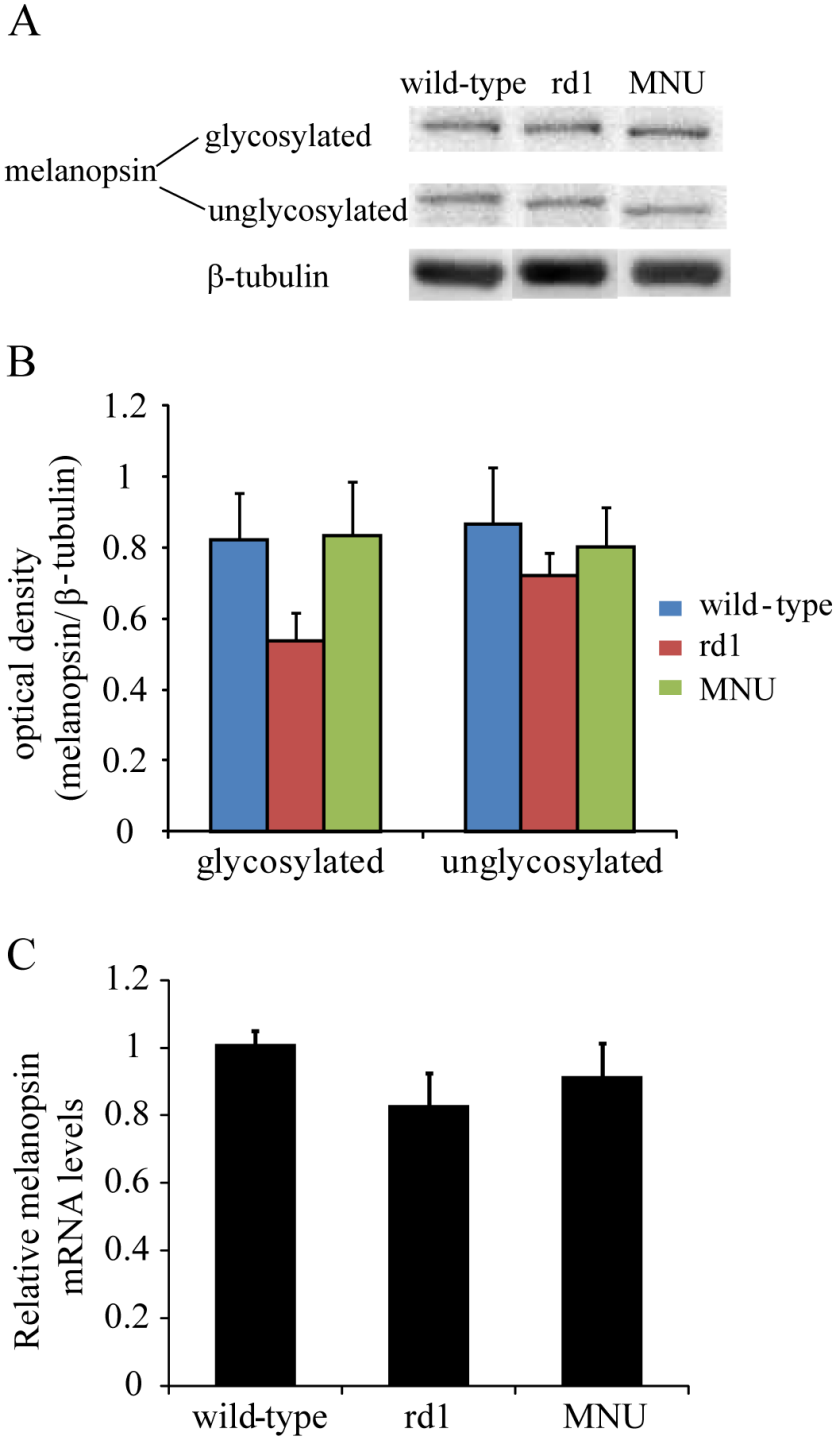
Melanopsin expression levels in retina were unaltered following photoreceptor loss in both rd1 and MNU-injected mice. (A) Representative immunoblots for melanopsin and the corresponding β-tubulin for wild-type, rd1 and MNU-injected mice. Melanopsin immunoblot produced two bands: glycosylated protein at 85 kDa and unglycosylated protein at 53 kDa. (B) Optical density ratio (melanopsin to β-tubulin) for glycosylated and unglycosylated melanopsin (mean±SE). The levels of glycosylated and unglycosylated melanopsin in rd1 (0.54 ± 0.08 and 0.72 ± 0.07, respectively) and MNU-injected mice (0.84 ± 0.15 and 0.8 ± 0.11) were statistically similar to those in wild-type (0.82 ± 0.13 and 0.86 ± 0.16) (F_2,9_ = 1.8, *P* = 0.22 and F_2,9_ = 0.36, *P* = 0.7, respectively; one-way ANOVA; n = 4 mice). (C) Relative levels of melanopsin mRNA in rd1 (0.83 ± 0.09) and MNU-injected mice (0.92 ± 0.1) were statistically similar to those in wild-type (1.01 ± 0.04) (F_2,30_ = 1.25, *P* = 0.3; one-way ANOVA; n = 11 mice).

**Fig 6:**
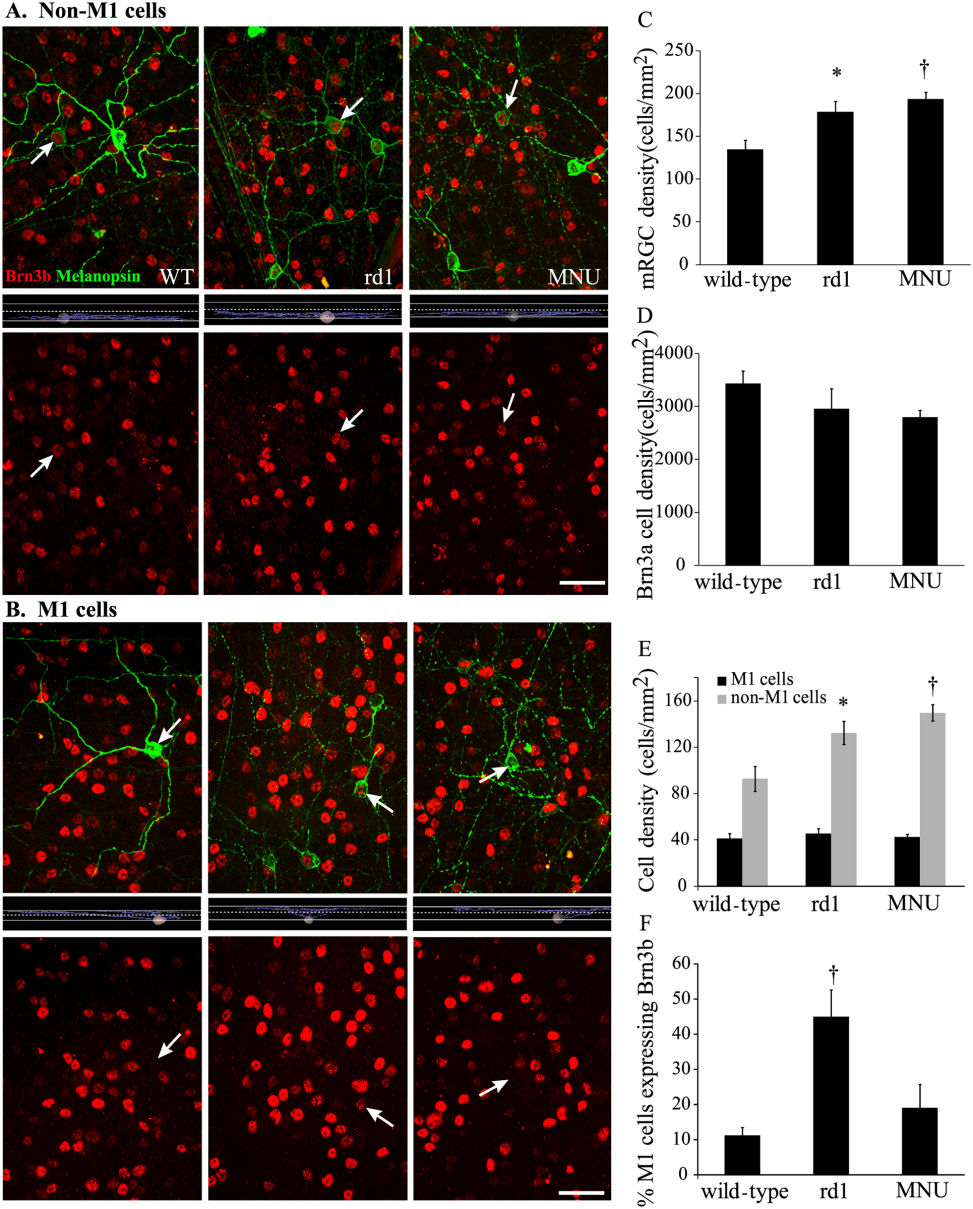
Both rd1 and MNU-injected mice had higher numbers of mRGCs, but only rd1 mice had a higher proportion of Brn3b-expressing M1 cells than in wild-type. **A)** Representative images from flatmounted retinas double-labeled for melanopsin (*green*) and Brn3b (*red*), showing non-M1 cells (*arrows*) in wild-type (WT, *left*), rd1 (*middle*) and MNU (7 days post injection; *right*). Top row shows original images, middle row shows dendritic stratification of the marked non-M1 (*arrow*) cells, and bottom row shows the same images with melanopsin signal digitally removed to more clearly show the Brn3b labeling in the non-M1 cells. The middle images show 2 thin solid lines marking the boundaries of the IPL and a thin dashed line separating the Off (top) and On (bottom) sublaminas in the IPL. The faintly-labeled non-M1 cells (*arrows*) stratified in ON sublamina and expressed Brn3b in wild-type, rd1 and MNU-injected mice. Scale bar: 50 μm. **B)** Representative images from flatmounted retinas double-labeled for melanopsin (*green*) and Brn3b (*red*), showing M1 cells (*arrows*) in wild-type (*left*), rd1 (*middle*) and MNU (7 days post injection; *right*). Top row shows original images, middle row shows dendritic stratification of the marked M1 (*arrow*) cells, and bottom row shows the same images with melanopsin signal digitally removed to more clearly show the Brn3b labeling in the M1 cells. The middle images show 2 thin solid lines marking the boundaries of the IPL and a thin dashed line separating the Off (top) and On (bottom) sublaminas in the IPL. The brightly-labeled M1 cells (*arrows*) stratified in OFF sublamina and expressed Brn3b in rd1, but not in wild-type or MNU-injected mice. Scale bar: 50 μm. **C)** The density of mRGCs (mean ± SE) in wild-type, rd1 and MNU-injected mice. A one-way ANOVA showed significant differences among the 3 groups (F_2,33_ = 8.18; *P* = 0.001; n = 8 mice for wild-type and rd1, n = 20 for MNU-injected). A post hoc Holm-Sidak test revealed that both rd1 and MNU-injected mice had significantly higher numbers of mRGCs than wild-type. *P <0.05; ^†^*P* <0.001. **D)** The numbers of non-mRGCs, identified by presence of Brn3a immunoreactivity, in wild-type, rd1, and MNU-injected mice were statistically similar (F_2,5_ = 1.16; *P* = 0.39; one-way ANOVA; n = 3 mice for wild-type and rd1, n = 2 for MNU-injected). **E)** Densities of M1 type of mRGCs in wild-type, rd1 and MNU-injected mice were similar (F_2,33_ = 0.32, *P* = 0.73; one-way ANOVA), whereas the densities of non-M1 cells were different (F_2,33_ = 9.74, *P* <0.001). A post hoc Holm-Sidak test showed that both rd1 and MNU-injected mice had significantly higher numbers of non-M1 cells than in wild-type (n = 8 mice for wild-type and rd1, n = 20 for MNU-injected). The MNU-injected group included animals in which mRGCs were counted at 3, 7, 14 or 28 days after the MNU injection (n = 5 mice each); data from all samples were pooled, because there was no statistical difference among the subgroups (data not shown). *P <0.05, †*P* <0.001. **F)** There was a significant difference in the proportion of M1 cells that expressed Brn3b among wild-type, rd1 and MNU-injected mice (F_2,32_ = 13.39, *P* <0.001, one-way ANOVA; n = 8 mice for wild-type, 7 for rd1, 20 for MNU). A post hoc Holm-Sidak test showed that the numbers in rd1 mice, but not in MNU-injected mice, were significantly higher than in wild-type. †*P* <0.001.

If classical photoreceptors are responsible for PLR even at high irradiances, and considering that the extent of photoreceptor loss in rd1 mouse is similar (m-cones) or more (rods, s-cones) than in MNU-injected mouse, it was unclear why the high-irradiance PLR is intact in rd1 mouse. One possibility was that loss of classical photoreceptors during development in rd1 mouse results in a compensatory response in mRGCs which may then take over the PLR function at high irradiances. To explore this, we measured melanopsin expression levels (Fig 5) and counted the numbers of mRGCs in wild-type, rd1 and MNU-injected mice (Fig 6).

Using quantitative immunoblotting and real-time PCR, we measured melanopsin protein and mRNA levels respectively in wild-type, rd1 and MNU-injected (14 dpi) mice. We found that melanopsin expression levels (protein and mRNA) in both rd1 and MNU-injected mice were statistically similar to those in wild-type (Fig 5). However, the numbers of mRGCs in both rd1 and MNU-injected mice were significantly higher than in wild-type (Fig 6C). To investigate whether this effect was specific to mRGCs, we also counted the non-mRGCs using Brn3a immunohistochemistry. The transcription factor Brn3a has been shown to be absent in mRGCs [9,36]. We found that the numbers of Brn3a-positive RGCs in rd1 and MNU-injected mice were not statistically different from wild-type (Fig 6D). These results suggested that loss of photoreceptors, irrespective of the etiology and the age of onset, leads to increase in the numbers of mRGCs.

The mRGCs have been classified based on their dendritic stratification into M1 and non-M1 types (Fig 6A and 6B) [9,19,31,37]. Nearly all non-M1 and a small subset (10%–15%) of M1 cells express the transcription factor Brn3b, while the majority of M1 cells do not [9,38]. The Brn3b-expressing mRGCs have been implicated in PLR [25], although it is not clear whether Brn3b-expressing M1 cells, non-M1 cells, or both are involved. To investigate whether the increase in the numbers of mRGCs was specific to a subtype, we counted M1 and non-M1 cells identified based on their dendritic stratification in the IPL [9].

We found that the densities of M1 cells in rd1 (mean ± SD; 45 ± 12 cells/mm^2^) and MNU-injected mice (43 ± 10) were similar to that in wild-type (41 ± 12) (Fig 6E). However, the density of non-M1 cells was significantly greater in both rd1 (132 ± 28) and MNU-injected mice (150 ± 32) compared with wild-type (93 ± 31) (Fig 6E). Since the number of non-M1 cells, which express Brn3b, increased after loss of classical photoreceptor, we asked if the Brn3b-expressing M1 cells were also affected in the two models. Interestingly, we found that 42% of the M1 cells (19 ± 7 out of 45 ± 12 cells/mm^2^; mean ± SD) expressed Brn3b in rd1 mouse, a proportion that was approximately four-fold higher than in wild-type (11%, or 4.6 ± 3 out of 41 ± 12) (Fig 6F). However, the proportion of these cells in MNU-injected mouse (19%, or 8 ± 5 out of 43 ± 10) was statistically similar to that in wild-type (Fig 6F). Even in the mouse injected with NaIO_3_ during adulthood, the proportion of Brn3b-expressing M1 cells (5%; n = 3 mice) was statistically similar to that in wild-type mouse (*P* = 0.6; data not illustrated).

### Classical photoreceptor degeneration induced during development resulted in partial rescue of PLR at high irradiances

**Fig 7:**
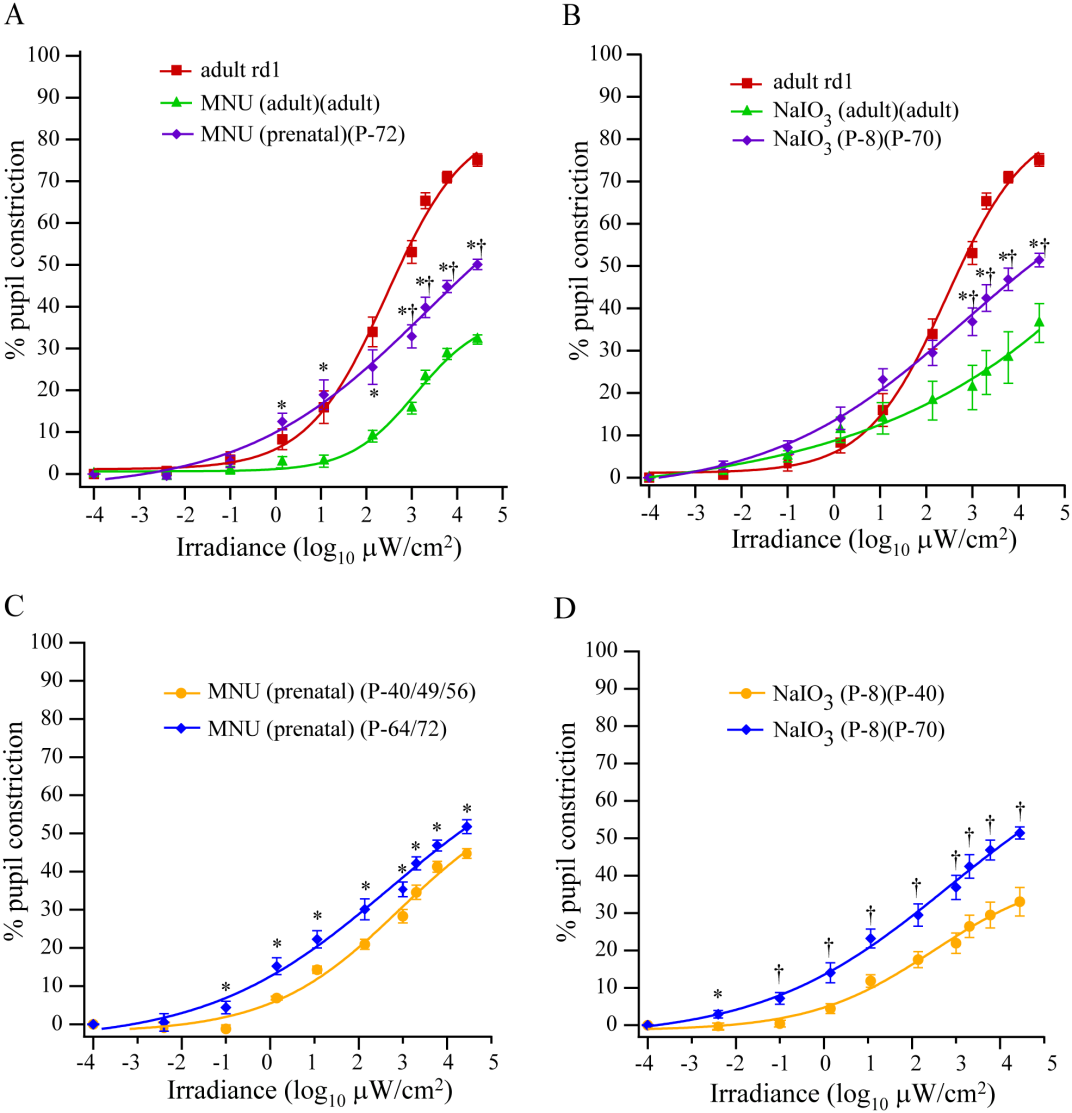
Photoreceptor loss induced during development resulted in partial rescue of PLR at higher irradiances. **A)** PLR in adult (P-72) mice that were injected with MNU prenatally (5 mg/kg i/p at embryonic day 16; n = 5 mice). The 2 parentheses in the label show animal ages when the drug was injected and PLR was recorded, respectively. PLRs of mice injected with MNU during adulthood and of adult rd1 mice are replotted from Fig 1B for comparison. A mixed ANOVA with repeated measures on irradiance showed significant differences among the 3 groups (F_2,18_ = 60.7, *P* <<0.0001). A post hoc test using Bonferroni correction showed that the PLR in MNU (prenatal)(P-72) animals was significantly higher than in MNU (adult)(adult) animals (*P* = 0.0002), but significantly lower than the adult rd1 mice (*P* = 0.001). A pairwise comparison at each irradiance revealed that the PLR in MNU (prenatal)(P-72) mice was similar to that in MNU (adult)(adult) at <1 μW/cm^2^, but significantly higher at irradiances >1 μW/cm^2^. The PLR in MNU (prenatal)(P-72) mice was similar to that in adult rd1 mice at <10^3^ μW/cm^2^, but significantly lower at irradiances >10^3^ μW/cm^2^. * *P* ≤0.005 for MNU (prenatal) versus MNU (adult); ^†^*P* <0.0001 for MNU (prenatal) versus adult rd1. **B)** PLR in adult (P-70) mice that were injected with NaIO_3_ at P-8 (60 mg/kg; i/p; n = 6 mice). PLRs of mice injected with NaIO_3_ during adulthood and of adult rd1 mice are replotted from Figs 2A and 1B, respectively, for comparison. A mixed ANOVA with repeated measures on irradiance showed significant differences among the 3 groups (F_2,17_ = 14.3; *P* = 0.0003). A post hoc test using Bonferroni correction showed that the PLR in both rd1 and NaIO_3_ (P-8)(P-70) mice was significantly higher than the NaIO_3_ (adult)(adult) mice (*P* = 0.0002 and 0.038, respectively), while the PLR in rd1 mice was similar to that in NaIO_3_ (P-8)(P-70) mice (*P* = 0.098). Pairwise comparisons at individual irradiances revealed that PLR was similar in all 3 groups at irradiances <10^3^ μW/cm^2^. However, at >10^3^ μW/cm^2^, the PLR in NaIO_3_ (P-8)(P-70) mice was significantly higher than in NaIO_3_ (adult)(adult) mice (*P* = 0.012 at 10^3^ μW/cm^2^ to *P* = 0.002 at the highest irradiance) but significantly lower than in rd1 mice (*P* = 0.006 at 10^3^ μW/cm^2^ to *P* = 0.00001 at the highest irradiance). * *P* ≤0.05 for NaIO_3_ (P-8)(P-70) versus NaIO_3_ (adult)(adult); ^†^*P* <0.005 for NaIO_3_ (P-8)(P-70) versus rd1 mice. **C)** In mice injected prenatally with MNU, the PLR recorded during adulthood (P-64 or P-72) was significantly higher than during development (P-40, P-49, or P-56) (F_1,8_ = 12.6, *P* = 0.008, n = 5 mice; two-way ANOVA with repeated measures). A pairwise comparison revealed significant differences at irradiances ≥0.1 μW/cm^2^. *P <0.05. **D)** In mice injected at P-8 with NaIO_3_, the PLR recorded during adulthood (P-70) was significantly higher than during development (P-40) (F_1,10_ = 18.02, *P* = 0.002; n = 6 mice; two-way ANOVA with repeated measures). A pairwise comparison revealed significant differences at all irradiances. *P <0.05; ^†^*P* <0.01.

If the developmental loss of classical photoreceptors was responsible for the intact high-irradiance PLR in rd1 mouse, we expected that photoreceptor degeneration induced with MNU or NaIO_3_ during development will have a similar outcome. To test this, we injected MNU prenatally (i/p to pregnant mothers) [28]; the pups were reared normally till adulthood and their PLR was recorded at regular intervals from P-40 to P-72. The PLR in the prenatally-injected animals at P-72 was significantly higher than in the mice injected with MNU during adulthood at medium-to-high (≥1 μW/cm^2^) irradiances, but significantly lower than in the adult rd1 mice at high (≥10^3^ μW/cm^2^) irradiances (Fig 7A).

In another experiment, we injected NaIO_3_ in wild-type mice at P-8, the time when photoreceptors start to degenerate in rd1 mouse. PLR was recorded in these mice at regular intervals from P-40 to P-70. At low-to-medium irradiances (<10^3^ μW/cm^2^), the PLR in these mice at P-70 was statistically similar to that in mice injected with NaIO_3_ during adulthood and that in adult rd1 mice (Fig 7B). However, at high irradiances (≥10^3^ μW/cm^2^), PLR in these mice was significantly higher than in mice injected with NaIO_3_ during adulthood and significantly lower than in adult rd1 mice (Fig 7B).

Interestingly, in the mice injected with MNU or NaIO_3_ during development, the high-irradiance PLR increased over time (Fig 7C and 7D). Overall, these results suggested that loss of photoreceptors during development leads to a compensatory increase in high-irradiance PLR.

### Classical photoreceptor loss induced during development resulted in a higher proportion of Brn3b-expressing M1 cells

**Fig 8:**
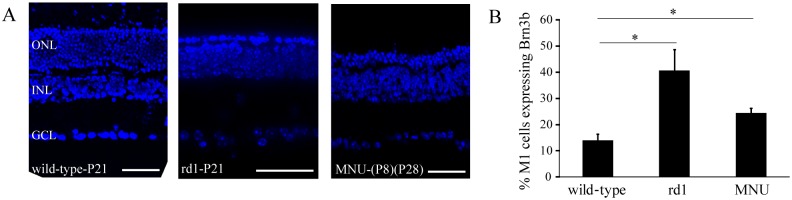
Similarly to rd1 mouse, MNU-induced photoreceptor loss during development resulted in a higher proportion of Brn3b-expressing M1 cells. **A)** DAPI-labeled retinal sections of P21 wild-type mouse (*left*), P21 rd1 mouse (*middle*) and P28 mouse injected with MNU at P8 (*right*) showing the difference in numbers of cell layers in ONL. Scale bar: 50 μm. **B)** Proportion of Brn3b-expressing M1 cells in P-22 to P-30 mice in which MNU was injected at P-8 to P-12 was compared with that in age-matched wild-type and rd1 mice (both P-21). A Welch ANOVA showed significant differences among the groups (F_2,8.6_ = 8.17; *P* = 0.01; n = 6 mice), and a post-hoc Games-Howell test revealed that both rd1 and MNU-injected mice had significantly higher proportions of Brn3b-expressing M1 cells than in wild-type (*P* = 0.03 and 0.02, respectively). *P <0.05.

To investigate whether photoreceptor degeneration induced during development would also result in more M1 cells expressing Brn3b, we injected MNU (60 mg/kg) in wild-type mice at P-8 to P-12, the time when photoreceptors start to degenerate in rd1 mouse. At 2–3 weeks after the injection, only 1–3 cell layers remained in the ONL, similar to age-matched rd1 (P-21) mouse (Fig 8A). The proportion of M1 cells that expressed Brn3b in these animals (24% or 13 ± 4 out of 53 ± 18 cells/mm^2^; mean ± SD) as well as in age-matched rd1 mice (41% or 18 ± 10 cells/mm^2^ out of 44 ± 17) was significantly higher than in age-matched wild-type mouse (14% or 6 ± 2 cells/mm^2^ out of 43 ± 11), while there was no statistical difference between the MNU-injected and rd1 mice (Fig 8B).

## Discussion

### Primary role for classical photoreceptors in generating PLR

PLR is thought to originate from classical photoreceptors at low irradiances and from melanopsin activation at high irradiances [1,7,21–23]. However, since classical photoreceptors can encode all physiologically-relevant light levels [39], it seemed puzzling that melanopsin activation should contribute to normal PLR. By inducing photoreceptor loss after normal retinal development in mice, we show here that classical photoreceptors are required for PLR at all irradiances. These mice showed no pupil constriction at low irradiances, and a severely attenuated response at high irradiances. Using two experimental approaches we demonstrate that the loss of PLR in these mice was attributable to loss of photoreceptors (Fig 2).

The inducible mouse models for photoreceptor loss used in this study have been well characterized. MNU is one of the most commonly used chemical agents to induce photoreceptor degeneration [27,40,41]. Multiple approaches, such as biochemical assays, electroretinogram measurements, and behavioral tests have demonstrated that both rods and cones are severely impaired and gross vision is lost in both MNU- and NaIO_3_-treated animals [27,42–44]. However, the ganglion cell layer is relatively preserved in the MNU mouse model [27] and has not been investigated in the NaIO_3_ model. Our finding that MNU did not alter the PLR in rd1 mouse, further confirms that MNU does not adversely affect the retinal ganglion cells, especially the mRGCs (Fig 2A).

Several observations suggested that the residual PLR at high irradiances in the MNU-injected mouse originated from the remnant classical photoreceptors. First, MNU-injected mouse exhibited a transient PLR response which was absent in rd1 mouse. Second, injecting NaIO_3_ in the MNU-injected mouse resulted in additional reduction in PLR. Finally, the numbers of remnant rods and s-cones in MNU-injected mouse were considerably higher than in rd1 mouse. Rods, although they operate at low light levels, have been recently shown to also respond to very bright light [45,46]. Interestingly, s-cones have been shown in mouse to produce a sustained response to bright light in olivary pretectal nucleus (OPN), the brain area responsible for PLR [6]. A previous report, showing that MNU-injected mouse retina retains some light response, provides evidence that remnant photoreceptors in this model can not only respond to light, but also send signals through the retina [47].

A relatively small component (up to ~15%) of the high-irradiance PLR also originated from activation of melanopsin. We concluded this from the extent of PLR reduction observed in the mice with both MNU and NaIO_3_ injected and melanopsin pharmacologically blocked (Fig 4). This magnitude of PLR reduction was consistent with previous observations in Opn4^-/-^ mouse [21,22]. Even if the antagonist did not block melanopsin activation completely, the melanopsin contribution to total PLR could not have exceeded ~25% at the highest irradiance used here [24]. Furthermore, the high irradiances (≥10^3^ μW/cm^2^) where melanopsin did contribute to PLR are likely non-physiological for a mouse, a nocturnal animal. Using a genetic mouse model where m-cones respond to a higher wavelength (650 nm), Lall et al. reported that melanopsin contribution to high-irradiance PLR is greater than that of m-cones, but they also showed that 40% of pupil constriction could be achieved at high irradiances in cone-only (Gnat^-/-^,Opn4^-/-^) mouse [5], suggesting a significant role for cones at high irradiances. The melanopsin contribution to PLR may vary depending on stimulation conditions, such as stimulus wavelength, stimulus duration, and light adaption [5,48,49].

### Photoreceptor loss during development results in compensatory rescue of high-irradiance PLR

If classical photoreceptors are responsible for PLR at all irradiances, it was unclear how the PLR is preserved at high irradiances in rd1 mouse. The remnant photoreceptors cannot explain this, considering that rd1 mouse has fewer photoreceptors than MNU-injected mouse. We explored the possibility that the loss of photoreceptors during development in rd1 mouse results in some compensatory response in mRGCs.

Ablating the mRGCs abolishes PLR and circadian photoentrainment [50,51]. Ablating the Brn3b-expressing mRGCs results in severely attenuated PLR, implying that PLR is mediated specifically by these cells [25]. However, since Brn3b is expressed by both M1 and non-M1 type of mRGCs, it was not clear which of these cell types mediate PLR. We found that in rd1 mouse, in which the photoreceptors start to degenerate during development, the numbers of the Brn3b-expressing M1 cells were higher than in mice in which photoreceptor loss was induced during adulthood. Since the high-irradiance PLR is preserved in rd1 mouse but not in MNU-injected mouse, this suggested that the increased number of Brn3b-expressing M1 cells is responsible for the preserved PLR. Consistent with this, we found that inducing photoreceptor degeneration with MNU or NaIO_3_ during development also resulted in significantly higher proportions of Brn3b-expressing M1 cells and partially rescued PLR. Interestingly, onset of PLR during development in wild-type mouse is marked by the innervation of OPN shell by M1 cells [52]. The higher numbers of Brn3b-expressing M1 cells following developmental loss of photoreceptors (either in rd1 mouse or in inducible models) could potentially cause enhanced innervation of OPN by these cells, leading in turn to the rescued high-irradiance PLR in these mice.

The photoreceptor loss induced during development, however, led to only a partially-rescued high-irradiance PLR. One possibility was that the higher PLR in these mice at high irradiances originated from the remnant photoreceptors (Fig 3) and not from any compensatory response in mRGCs. However, our finding that the PLR in these mice increased over time during development (Fig 7C and 7D) strongly suggested that the rescued high-irradiance PLR was an emergent property that cannot be explained by remnant photoreceptors.

### Regulation of mRGCs by photoreceptors

A previous report showed that rd1 mouse has more mRGCs than the congenic wildtype; the reason they suggested was that the mRGCs in rd1 mouse do not undergo apoptosis during development [23]. However, our finding that the numbers of mRGCs was higher even when photoreceptor degeneration was induced with MNU during adulthood suggests alternative/additional mechanisms. Although both M1 and non-M1 type of mRGCs receive signals from the photoreceptors, the latter receive a stronger input [53,54]. We found that loss of photoreceptors resulted in increased numbers of non-M1 cells in both rd1 and MNU-injected mice (Fig 6E), indicating that photoreceptors regulate the expression of melanopsin in RGCs, dynamically and negatively. It should also be noted that the numbers of mRGCs in rd/rd cl mouse have been reported to be similar to its congenic control [55–57], possibly because the loss of photoreceptors occurs at a much faster rate in this model.

Despite the higher number of mRGCs in both rd1 and MNU-injected mice, we did not find any change in melanopsin expression (protein or m-RNA) levels (Fig 5). This is consistent with previous reports showing no change in melanopsin expression in rd/rd cl or rd1 mouse models [23,55]. However, melanopsin levels have been reported to be reduced in rat models of retinal degeneration [32,58,59], raising the possibility that different mechanisms may be involved in different species.

The number of Brn3b-expressing RGCs declines during early development (from E16 to P-5) in normal mouse [60]. It is possible that Brn3b expression by RGCs is also negatively regulated by photoreceptors during development. This was consistent with our observation that the numbers of Brn3b-expressing M1 cells declined from P-14 to adulthood in wild-type, but not in rd1 mouse (data not shown). This could explain the higher numbers of Brn3b-expressing M1 cells in rd1 mice or in mice in which the photoreceptor degeneration is induced during development than in wild-type mice or in mice in which the photoreceptor degeneration is induced during adulthood.

## References

[1] Hattar S, Lucas RJ, Mrosovsky N, Thompson S, Douglas RH, Hankins MW, et al Melanopsin and rod-cone photoreceptive systems account for all major accessory visual functions in mice. Nature. 2003;424(6944):76–81. 10.1038/nature01761 12808468 PMC2885907

[2] Thompson S, Mullins RF, Philp AR, Stone EM, Mrosovsky N. Divergent phenotypes of vision and accessory visual function in mice with visual cycle dysfunction (Rpe65 rd12) or retinal degeneration (rd/rd). Investigative ophthalmology & visual science. 2008;49(6):2737–42. 10.1167/iovs.07-1546 . 18515598

[3] Altimus CM, Guler AD, Alam NM, Arman AC, Prusky GT, Sampath AP, et al Rod photoreceptors drive circadian photoentrainment across a wide range of light intensities. Nature neuroscience. 2010;13(9):1107–12. 10.1038/nn.2617 20711184 PMC2928860

[4] Ecker JL, Dumitrescu ON, Wong KY, Alam NM, Chen SK, LeGates T, et al Melanopsin-expressing retinal ganglion-cell photoreceptors: cellular diversity and role in pattern vision. Neuron. 2010;67(1):49–60. 10.1016/j.neuron.2010.05.023 20624591 PMC2904318

[5] Lall GS, Revell VL, Momiji H, Al Enezi J, Altimus CM, Guler AD, et al Distinct contributions of rod, cone, and melanopsin photoreceptors to encoding irradiance. Neuron. 2010;66(3):417–28. 10.1016/j.neuron.2010.04.037 20471354 PMC2875410

[6] Allen AE, Brown TM, Lucas RJ. A distinct contribution of short-wavelength-sensitive cones to light-evoked activity in the mouse pretectal olivary nucleus. The Journal of neuroscience: the official journal of the Society for Neuroscience. 2011;31(46):16833–43. 10.1523/JNEUROSCI.2505-11.2011 22090509 PMC3245852

[7] Thompson S, Stasheff SF, Hernandez J, Nylen E, East JS, Kardon RH, et al Different inner retinal pathways mediate rod-cone input in irradiance detection for the pupillary light reflex and regulation of behavioral state in mice. Investigative ophthalmology & visual science. 2011;52(1):618–23. 10.1167/iovs.10-6146 20847113 PMC3053302

[8] Estevez ME, Fogerson PM, Ilardi MC, Borghuis BG, Chan E, Weng S, et al Form and function of the M4 cell, an intrinsically photosensitive retinal ganglion cell type contributing to geniculocortical vision. The Journal of neuroscience: the official journal of the Society for Neuroscience. 2012;32(39):13608–20. 10.1523/JNEUROSCI.1422-12.2012 23015450 PMC3474539

[9] Jain V, Ravindran E, Dhingra NK. Differential expression of Brn3 transcription factors in intrinsically photosensitive retinal ganglion cells in mouse. The Journal of comparative neurology. 2012;520(4):742–55. 10.1002/cne.22765 . 21935940

[10] Gerlai R. Gene-targeting studies of mammalian behavior: is it the mutation or the background genotype? Trends in neurosciences. 1996;19(5):177–81. . 8723200 10.1016/s0166-2236(96)20020-7

[11] Picciotto MR, Wickman K. Using knockout and transgenic mice to study neurophysiology and behavior. Physiological reviews. 1998;78(4):1131–63. . 9790572 10.1152/physrev.1998.78.4.1131

[12] Gerlai R. Gene targeting: technical confounds and potential solutions in behavioral brain research. Behavioural brain research. 2001;125(1–2):13–21. . 11682088 10.1016/s0166-4328(01)00282-0

[13] Greenspan RJ. The flexible genome. Nature reviews Genetics. 2001;2(5):383–7. 10.1038/35072018 . 11331904

[14] Marder E, Goaillard JM. Variability, compensation and homeostasis in neuron and network function. Nature reviews Neuroscience. 2006;7(7):563–74. 10.1038/nrn1949 . 16791145

[15] Dkhissi-Benyahya O, Gronfier C, De Vanssay W, Flamant F, Cooper HM. Modeling the role of mid-wavelength cones in circadian responses to light. Neuron. 2007;53(5):677–87. 10.1016/j.neuron.2007.02.005 17329208 PMC1950159

[16] Ostergaard J, Hannibal J, Fahrenkrug J. Synaptic contact between melanopsin-containing retinal ganglion cells and rod bipolar cells. Investigative ophthalmology & visual science. 2007;48(8):3812–20. 10.1167/iovs.06-1322 . 17652756

[17] Viney TJ, Balint K, Hillier D, Siegert S, Boldogkoi Z, Enquist LW, et al Local retinal circuits of melanopsin-containing ganglion cells identified by transsynaptic viral tracing. Current biology: CB. 2007;17(11):981–8. 10.1016/j.cub.2007.04.058 . 17524644

[18] Wong KY, Dunn FA, Graham DM, Berson DM. Synaptic influences on rat ganglion-cell photoreceptors. The Journal of physiology. 2007;582(Pt 1):279–96. 10.1113/jphysiol.2007.133751 17510182 PMC2075299

[19] Schmidt TM, Taniguchi K, Kofuji P. Intrinsic and extrinsic light responses in melanopsin-expressing ganglion cells during mouse development. Journal of neurophysiology. 2008;100(1):371–84. 10.1152/jn.00062.2008 18480363 PMC2493479

[20] Weng S, Estevez ME, Berson DM. Mouse ganglion-cell photoreceptors are driven by the most sensitive rod pathway and by both types of cones. PloS one. 2013;8(6):e66480 10.1371/journal.pone.0066480 23762490 PMC3676382

[21] Lucas RJ, Hattar S, Takao M, Berson DM, Foster RG, Yau KW. Diminished pupillary light reflex at high irradiances in melanopsin-knockout mice. Science. 2003;299(5604):245–7. 10.1126/science.1077293 . 12522249

[22] Panda S, Provencio I, Tu DC, Pires SS, Rollag MD, Castrucci AM, et al Melanopsin is required for non-image-forming photic responses in blind mice. Science. 2003;301(5632):525–7. 10.1126/science.1086179 . 12829787

[23] Ruggiero L, Allen CN, Brown RL, Robinson DW. The development of melanopsin-containing retinal ganglion cells in mice with early retinal degeneration. The European journal of neuroscience. 2009;29(2):359–67. 10.1111/j.1460-9568.2008.06589.x 19200239 PMC2764118

[24] Jones KA, Hatori M, Mure LS, Bramley JR, Artymyshyn R, Hong SP, et al Small-molecule antagonists of melanopsin-mediated phototransduction. Nature chemical biology. 2013;9(10):630–5. 10.1038/nchembio.1333 23974117 PMC3839535

[25] Chen SK, Badea TC, Hattar S. Photoentrainment and pupillary light reflex are mediated by distinct populations of ipRGCs. Nature. 2011;476(7358):92–5. 10.1038/nature10206 21765429 PMC3150726

[26] Enzmann V, Row BW, Yamauchi Y, Kheirandish L, Gozal D, Kaplan HJ, et al Behavioral and anatomical abnormalities in a sodium iodate-induced model of retinal pigment epithelium degeneration. Experimental eye research. 2006;82(3):441–8. 10.1016/j.exer.2005.08.002 . 16171805

[27] Nagar S, Krishnamoorthy V, Cherukuri P, Jain V, Dhingra NK. Early remodeling in an inducible animal model of retinal degeneration. Neuroscience. 2009;160(2):517–29. 10.1016/j.neuroscience.2009.02.056 . 19272416

[28] Smith SB, Yielding KL. Retinal degeneration in the mouse. A model induced transplacentally by methylnitrosourea. Experimental eye research. 1986;43(5):791–801. . 3803463 10.1016/s0014-4835(86)80010-0

[29] Aleman TS, Jacobson SG, Chico JD, Scott ML, Cheung AY, Windsor EA, et al Impairment of the transient pupillary light reflex in Rpe65(-/-) mice and humans with leber congenital amaurosis. Investigative ophthalmology & visual science. 2004;45(4):1259–71. . 15037595 10.1167/iovs.03-1230

[30] Panda S, Sato TK, Castrucci AM, Rollag MD, DeGrip WJ, Hogenesch JB, et al Melanopsin (Opn4) requirement for normal light-induced circadian phase shifting. Science. 2002;298(5601):2213–6. 10.1126/science.1076848 . 12481141

[31] Berson DM, Castrucci AM, Provencio I. Morphology and mosaics of melanopsin-expressing retinal ganglion cell types in mice. The Journal of comparative neurology. 2010;518(13):2405–22. 10.1002/cne.22381 20503419 PMC2895505

[32] Boudard DL, Mendoza J, Hicks D. Loss of photic entrainment at low illuminances in rats with acute photoreceptor degeneration. The European journal of neuroscience. 2009;30(8):1527–36. 10.1111/j.1460-9568.2009.06935.x . 19821841

[33] Dagar S, Nagar S, Goel M, Cherukuri P, Dhingra NK. Loss of photoreceptors results in upregulation of synaptic proteins in bipolar cells and amacrine cells. PloS one. 2014;9(3):e90250 10.1371/journal.pone.0090250 24595229 PMC3942420

[34] Goel M, Dhingra NK. Muller glia express rhodopsin in a mouse model of inherited retinal degeneration. Neuroscience. 2012;225:152–61. 10.1016/j.neuroscience.2012.08.066 . 22967839

[35] Lucas RJ, Douglas RH, Foster RG. Characterization of an ocular photopigment capable of driving pupillary constriction in mice. Nature neuroscience. 2001;4(6):621–6. 10.1038/88443 . 11369943

[36] Galindo-Romero C, Aviles-Trigueros M, Jimenez-Lopez M, Valiente-Soriano FJ, Salinas-Navarro M, Nadal-Nicolas F, et al Axotomy-induced retinal ganglion cell death in adult mice: quantitative and topographic time course analyses. Experimental eye research. 2011;92(5):377–87. 10.1016/j.exer.2011.02.008 . 21354138

[37] Baver SB, Pickard GE, Sollars PJ, Pickard GE. Two types of melanopsin retinal ganglion cell differentially innervate the hypothalamic suprachiasmatic nucleus and the olivary pretectal nucleus. The European journal of neuroscience. 2008;27(7):1763–70. 10.1111/j.1460-9568.2008.06149.x . 18371076

[38] Karnas D, Mordel J, Bonnet D, Pevet P, Hicks D, Meissl H. Heterogeneity of intrinsically photosensitive retinal ganglion cells in the mouse revealed by molecular phenotyping. The Journal of comparative neurology. 2013;521(4):912–32. 10.1002/cne.23210 . 22886938

[39] Umino Y, Solessio E, Barlow RB. Speed, spatial, and temporal tuning of rod and cone vision in mouse. The Journal of neuroscience: the official journal of the Society for Neuroscience. 2008;28(1):189–98. 10.1523/JNEUROSCI.3551-07.2008 18171936 PMC2847259

[40] Herrold KM. Pigmentary degeneration of the retina induced by N-methyl-N-nitrosourea. An experimental study in syrian hamsters. Archives of ophthalmology. 1967;78(5):650–3. . 6050851 10.1001/archopht.1967.00980030652017

[41] Nambu H, Yuge K, Nakajima M, Shikata N, Takahashi K, Miki H, et al Morphologic characteristics of N-methyl-N-nitrosourea-induced retinal degeneration in C57BL mice. Pathology international. 1997;47(6):377–83. . 9211525 10.1111/j.1440-1827.1997.tb04511.x

[42] Kiuchi K, Kondo M, Ueno S, Moriguchi K, Yoshizawa K, Miyake Y, et al Functional rescue of N-methyl-N-nitrosourea-induced retinopathy by nicotinamide in Sprague-Dawley rats. Current eye research. 2003;26(6):355–62. . 12868016 10.1076/ceyr.26.5.355.15435

[43] Kiuchi K, Yoshizawa K, Shikata N, Moriguchi K, Tsubura A. Morphologic characteristics of retinal degeneration induced by sodium iodate in mice. Current eye research. 2002;25(6):373–9. . 12789545 10.1076/ceyr.25.6.373.14227

[44] Machalinska A, Lubinski W, Klos P, Kawa M, Baumert B, Penkala K, et al Sodium iodate selectively injuries the posterior pole of the retina in a dose-dependent manner: morphological and electrophysiological study. Neurochemical research. 2010;35(11):1819–27. 10.1007/s11064-010-0248-6 20725778 PMC2957578

[45] Szikra T, Trenholm S, Drinnenberg A, Juttner J, Raics Z, Farrow K, et al Rods in daylight act as relay cells for cone-driven horizontal cell-mediated surround inhibition. Nature neuroscience. 2014;17(12):1728–35. 10.1038/nn.3852 . 25344628

[46] Allen AE, Cameron MA, Brown TM, Vugler AA, Lucas RJ. Visual responses in mice lacking critical components of all known retinal phototransduction cascades. PloS one. 2010;5(11):e15063 10.1371/journal.pone.0015063 21124780 PMC2993945

[47] Homma K, Osakada F, Hirami Y, Jin ZB, Mandai M, Takahashi M. Detection of localized retinal malfunction in retinal degeneration model using a multielectrode array system. Journal of neuroscience research. 2009;87(9):2175–82. 10.1002/jnr.22024 . 19224574

[48] Do MT, Yau KW. Adaptation to steady light by intrinsically photosensitive retinal ganglion cells. Proceedings of the National Academy of Sciences of the United States of America. 2013;110(18):7470–5. 10.1073/pnas.1304039110 23589882 PMC3645585

[49] Park JC, Moura AL, Raza AS, Rhee DW, Kardon RH, Hood DC. Toward a clinical protocol for assessing rod, cone, and melanopsin contributions to the human pupil response. Investigative ophthalmology & visual science. 2011;52(9):6624–35. 10.1167/iovs.11-7586 21743008 PMC3175993

[50] Guler AD, Ecker JL, Lall GS, Haq S, Altimus CM, Liao HW, et al Melanopsin cells are the principal conduits for rod-cone input to non-image-forming vision. Nature. 2008;453(7191):102–5. 10.1038/nature06829 18432195 PMC2871301

[51] Hatori M, Le H, Vollmers C, Keding SR, Tanaka N, Buch T, et al Inducible ablation of melanopsin-expressing retinal ganglion cells reveals their central role in non-image forming visual responses. PloS one. 2008;3(6):e2451 10.1371/journal.pone.0002451 18545654 PMC2396502

[52] McNeill DS, Sheely CJ, Ecker JL, Badea TC, Morhardt D, Guido W, et al Development of melanopsin-based irradiance detecting circuitry. Neural development. 2011;6:8 10.1186/1749-8104-6-8 21418557 PMC3070623

[53] Schmidt TM, Kofuji P. Differential cone pathway influence on intrinsically photosensitive retinal ganglion cell subtypes. The Journal of neuroscience: the official journal of the Society for Neuroscience. 2010;30(48):16262–71. 10.1523/JNEUROSCI.3656-10.2010 21123572 PMC3073605

[54] Zhao X, Stafford BK, Godin AL, King WM, Wong KY. Photoresponse diversity among the five types of intrinsically photosensitive retinal ganglion cells. The Journal of physiology. 2014 10.1113/jphysiol.2013.262782 . 24396062 PMC3979615

[55] Semo M, Lupi D, Peirson SN, Butler JN, Foster RG. Light-induced c-fos in melanopsin retinal ganglion cells of young and aged rodless/coneless (rd/rd cl) mice. The European journal of neuroscience. 2003;18(11):3007–17. . 14656296 10.1111/j.1460-9568.2003.03061.x

[56] Vugler A, Semo M, Ortin-Martinez A, Rojanasakul A, Nommiste B, Valiente-Soriano FJ, et al A role for the outer retina in development of the intrinsic pupillary light reflex in mice. Neuroscience. 2015;286:60–78. 10.1016/j.neuroscience.2014.11.044 . 25433236

[57] Semo M, Coffey P, Gias C, Vugler A. Retrograde Melanopsin Signaling Increases With Age in Retinal Degenerate Mice Lacking Rods and the Majority of Cones. Investigative ophthalmology & visual science. 2016;57(1):115–25. 10.1167/iovs.15-17609 . 26780315

[58] Wan J, Zheng H, Hu BY, Xiao HL, She ZJ, Chen ZL, et al Acute photoreceptor degeneration down-regulates melanopsin expression in adult rat retina. Neuroscience letters. 2006;400(1–2):48–52. 10.1016/j.neulet.2006.02.084 . 16580133

[59] Sakamoto K, Liu C, Tosini G. Classical photoreceptors regulate melanopsin mRNA levels in the rat retina. The Journal of neuroscience: the official journal of the Society for Neuroscience. 2004;24(43):9693–7. 10.1523/JNEUROSCI.2556-04.2004 . 15509757 PMC6730153

[60] Quina LA, Pak W, Lanier J, Banwait P, Gratwick K, Liu Y, et al Brn3a-expressing retinal ganglion cells project specifically to thalamocortical and collicular visual pathways. The Journal of neuroscience: the official journal of the Society for Neuroscience. 2005;25(50):11595–604. 10.1523/JNEUROSCI.2837-05.2005 . 16354917 PMC6726022

